# Ultrastructural Damages to H1N1 Influenza Virus Caused by Vapor Essential Oils

**DOI:** 10.3390/molecules27123718

**Published:** 2022-06-09

**Authors:** Valentina Noemi Madia, Walter Toscanelli, Daniela De Vita, Marta De Angelis, Antonella Messore, Davide Ialongo, Luigi Scipione, Valeria Tudino, Felicia Diodata D’Auria, Roberto Di Santo, Stefania Garzoli, Annarita Stringaro, Marisa Colone, Magda Marchetti, Fabiana Superti, Lucia Nencioni, Roberta Costi

**Affiliations:** 1 Dipartimento di Chimica e Tecnologie del Farmaco, Istituto Pasteur-Fondazione Cenci Bolognetti, Sapienza University of Rome, p.le Aldo Moro 5, I-00185 Rome, Italy; valentinanoemi.madia@uniroma1.it (V.N.M.); antonella.messore@uniroma1.it (A.M.); davide.ialongo@uniroma1.it (D.I.); luigi.scipione@uniroma1.it (L.S.); valeria.tudino@uniroma1.it (V.T.); roberto.disanto@uniroma1.it (R.D.S.); roberta.costi@uniroma1.it (R.C.); 2 Department of Public Health and Infectious Diseases, Laboratory Affiliated to Istituto Pasteur-Fondazione Cenci Bolognetti, Sapienza University of Rome, p.le Aldo Moro 5, I-00185 Rome, Italy; walter.toscanelli@uniroma1.it (W.T.); felicia.dauria@uniroma1.it (F.D.D.); lucia.nencioni@uniroma1.it (L.N.); 3 Department of Environmental Biology, Sapienza University of Rome, p.le Aldo Moro 5, I-00185 Rome, Italy; 4 Department of Drug Chemistry and Technologies, Sapienza University of Rome, p.le Aldo Moro 5, I-00185 Rome, Italy; stefania.garzoli@uniroma1.it; 5 National Center for Drug Research and Evaluation, Italian National Institute of Health, V.le Regina Elena, 299, I-00161 Rome, Italy; annarita.stringaro@iss.it (A.S.); marisa.colone@iss.it (M.C.); 6 National Centre for Innovative Technologies in Public Health, Italian National Institute of Health, V.le Regina Elena, 299, I-00161 Rome, Italy; magda.marchetti@iss.it (M.M.); fabiana.superti@iss.it (F.S.)

**Keywords:** influenza A H1N1 virus, essential oil vapors, bergamot, Chinese star anise, tea tree oil, eucalyptus, antivirals

## Abstract

Influenza viruses are transmitted from human to human via airborne droplets and can be transferred through contaminated environmental surfaces. Some works have demonstrated the efficacy of essential oils (EOs) as antimicrobial and antiviral agents, but most of them examined the liquid phases, which are generally toxic for oral applications. In our study, we describe the antiviral activity of *Citrus bergamia*, *Melaleuca alternifolia*, *Illicium verum* and *Eucalyptus globulus* vapor EOs against influenza virus type A. In the vapor phase, *C. bergamia* and *M. alternifolia* strongly reduced viral cytopathic effect without exerting any cytotoxicity. The *E. globulus* vapor EO reduced viral infection by 78% with no cytotoxicity, while *I. verum* was not effective. Furthermore, we characterized the EOs and their vapor phase by the head-space gas chromatography–mass spectrometry technique, observing that the major component found in each liquid EO is the same one of the corresponding vapor phases, with the exception of *M. alternifolia*. To deepen the mechanism of action, the morphological integrity of virus particles was checked by negative staining transmission electron microscopy, showing that they interfere with the lipid bilayer of the viral envelope, leading to the decomposition of membranes. We speculated that the most abundant components of the vapor EOs might directly interfere with influenza virus envelope structures or mask viral structures important for early steps of viral infection.

## 1. Introduction

Influenza viruses (Fam. Orthomyxoviridae) are etiological agents causing highly contagious acute respiratory syndromes and constituting a significant public health problem worldwide [1]. The virus is mainly transmitted from human to human through airborne droplets and may be transferred to a surface if it is touched by contaminated hands [2]. For these reasons, viral spread may be controlled by the disinfection of objects and sanitization of indoor air, and cleaning and disinfecting are considered an essential part of influenza control programs [3]. Indeed, it is well known that the microbial contamination of surfaces and medical devices, for example, plays an important role in the transmission of healthcare-associated pathogens [4]. Disinfection with chemical reagents is an important element of the biosecurity program against avian flu [5]. Even so, several issues are related with the use of chemical disinfectants such as cytotoxicity, corrosion and environmental toxicity. Phytochemicals, on the contrary, are an attractive source of environmental friendly, relatively inexpensive and available disinfectants [4]. Among them, various essential oils (EOs) from plants have been screened to assess their antimicrobial, antifungal, and antiviral activities [6]. Essential oils are complex mixtures of volatile compounds, such as monoterpenes, sesquiterpenes and phenylpropenes [7,8], which have been investigated for a wide range of properties [9,10,11].

Bergamot (*Citrus* × *bergamia* Risso & Poit.) belonging to *Rutaceae* family is classified as a hybrid of lemon (*Citrus limon* (L.) Osbeck) and bitter orange (*Citrus aurantium* L.). The plant is endemic in Calabria region (Italy) and is highly appreciated in the perfumery, cosmetic and food industries [12].

Chinese star anise (*Illicium verum* Hook. f.) is a tree of *Illiciaceae* family well known both for its EO used in liqueurs and the several medicinal properties, particularly in the traditional Chinese medicine [13].

*Melaleuca alternifolia* (Maiden & Betche) Cheel, most commonly known as tea tree oil (TTO), and *Eucalyptus globulus* Labill. (eucalyptus) belonging to the *Myrtaceae* family, are Australian endemic species. The leaves of both these plants are a source of EOs used for millenia as antiseptics and with antimicrobial activity against several respiratory tract pathogens, among others [14,15,16,17]. Interestingly, it was observed that bergamot, Chinese star anise, TTO and eucalyptus EOs inhibit influenza type A (H1N1) virus, and they have also been reported to be complementary and alternative treatment options for influenza infections [16].

Several studies have reported the antiviral activity of EOs against influenza A virus (IAV) [18,19,20,21,22,23,24,25,26], even though these works were carried out with the liquid phases of the oils and their components. On the other hand, it was demonstrated that the vapor phase of several EOs showed good antibacterial, antifungal and antiviral activity, sometimes better than the corresponding liquid phase of the oil [27,28,29,30]. Still, a paucity of data is reported in the literature on the anti-influenza effect of EO vapors. Indeed, the work of Vimalanathan and Hudson [31] is the only report describing the anti-influenza activity of vapor EOs. The authors deepened the activity against influenza virus of some EOs such as *C. bergamia* and *E. globulus* in their vapor phase without characterizing their chemical compositions. In the light of the above, we aimed at studying the activity of the vapors of EOs previously reported for their anti-influenza properties in their liquid phases [16,32,33]. Therefore, we tested bergamot, Chinese star anise, TTO and eucalyptus vapor essential oils for their anti-influenza activities. In addition, we carried out the phytochemical analysis of EOs in both their liquid and vapor phases to elucidate their chemical compositions by the head-space gas chromatography–mass spectrometry technique. This work is the first report describing the anti-influenza activity of EO vapors phytochemically analyzed in their vapor phase. Moreover, we deepened the mechanism of action of vapor EOs by checking the morphological integrity of virus particles through negative staining transmission electron microscopy, showing that they interfere with the lipid bilayer of the viral envelope, leading to the decomposition of membranes.

## 2. Results and Discussion

### 2.1. Headspace-Gas Chromatography/Mass Spectrometry (HS-GC/MS)

Essential oils were obtained from hydrodistillation with a yield of 0.9 and 4.7%.

Gas chromatographic analyses were carried out on the four EOs analyzed in this study both in their liquid and vapor phases. We analyzed the *C. bergamia* EO (BEO), *I. verum* EO (IV-EO), *M. alternifolia* (TTO), and *E. globulus* EO (EEO). The analysis of the liquid phase of EOs allowed to compare their chemical composition with that of their corresponding vapors.

Gas chromatographic analysis on liquid EOs led to the identification of 42 compounds in total listed in Table 1. Limonene (31.9%) and linalyl acetate (31.4%) followed by linalool (17.0%) were the main compounds found in *C. bergamia* EO. Anethole (93.0%), terpinen-4-ol (92.4%) and 1,8-cineole (93.7%) were the most abundant constituents in *I. verum*, *M. alternifolia* and *E. globulus* EOs, respectively. In general, terpenoids prevailed over sesquiterpenoids in all investigated EOs and represented the family of compounds prevalent except for *I. verum* EO where anethole, a non-terpene compound, was the one with the highest relative percentage. Results are in agreement with those reported in the literature [16,34,35,36].

The chemical composition of EOs in their vapor phase was described by the head-space GC/MS technique. Twenty-three compounds were identified and reported in Table 2. The volatile profile of EOs was dominated by terpenoids, while sesquiterpene compounds were missing. Limonene (51.2%) and β-pinene (20.4%) were the major compounds in *C. bergamia* EO. Anethole (49.1%) was the most abundant component even in the vapor phase of *I. verum* EO followed by α-pinene as the second principal molecule (19.1%). γ-Terpinene (29.3%) and 1,8-cineole (89.8%) dominated in *M. alternifolia* and *E. globulus* EOs, respectively. As observed by comparing Table 1 and Table 2, the major component found in each EO in its liquid phase is the same one present in the corresponding vapor phase, with the exception of *M. alternifolia*, where the main compound present in its liquid EO was the terpinen-4-ol, while the γ-terpinene was the most abundant in the corresponding vapor. Interestingly, the chemical composition of the *M. alternifolia* and *E. globulus* vapor EOs has been previously reported in the literature, and both analyses are in agreement with our results [37,38].

### 2.2. Antiviral Activity

**Figure 1:**
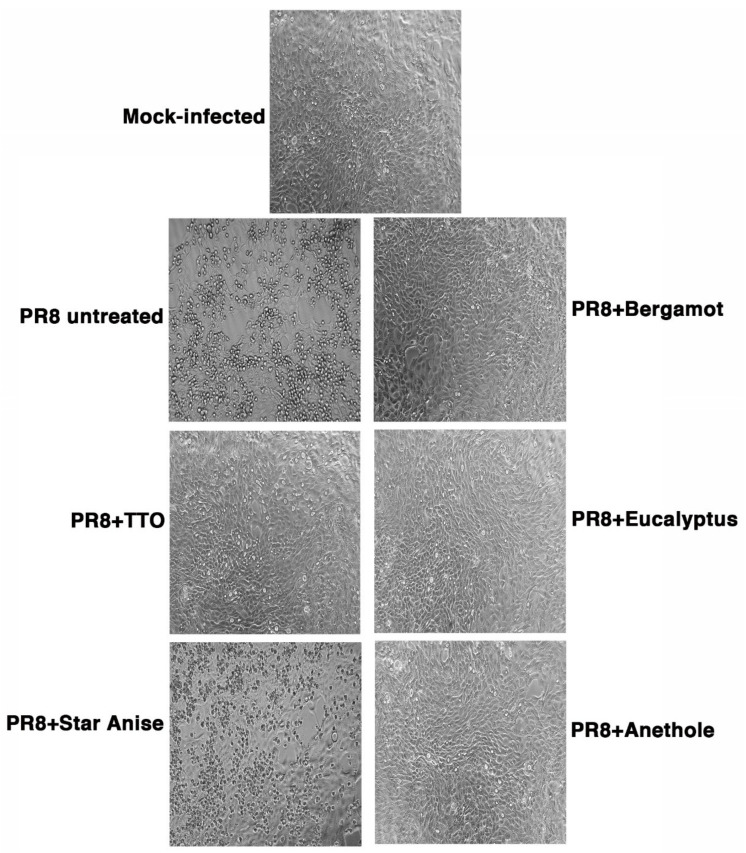
Cytopathic effect (CPE) induced by influenza virus treated with EOs vapor phases (PR8 + Bergamot; PR8 + TTO; PR8 + Eucalyptus; PR8 + Star Anise; PR8 + Anethole) or not treated (PR8 untreated) on MDCK cell monolayers analyzed by an inverted optical microscope after 24 h from infection. As control of infection, cells were mock-infected as described in methods.

As described in the methods, the virucidal activity was evaluated by exposing influenza A Puerto Rico 8/H1N1 virus (PR8) to vapor EOs for 30 min at 37 °C in an incubator at 5% CO_2_. Following the exposure to vapor phases, the virus was used to infect cell monolayers of Madin–Darby Canine Kidney (MDCK) cells. After 24 h infection, cells were observed under the microscope, and no cytopathic effect (CPE) was detectable in cells infected with the virus exposed to vapor bergamot and TTO EOs compared to cells infected with untreated viral suspension (PR8 untreated) (Figure 1). Similarly, no viral titer was quantified by Tissue Culture Infectious Dose 50% (TCID_50_), indicating a strong virucidal activity of both the vapor EOs, which was reflected in a sharp reduction in viral infectivity.

We also analyzed two other oil vapors, deriving from eucalyptus and Chinese star anise oils. As already described, PR8 was exposed to oil vapors for 30 min, and then, the virus used to infect cell monolayers for 24 h. As shown in Figure 1, while the eucalyptus vapor phase reduced CPE (viral titer measured by TCID_50_ was reduced by 78%: logTCID_50_ = 2.35 vs. logTCID_50_ = 1.68, PR8-untreated vs. -treated, respectively), Chinese star anise vapor was not effective. In particular, the virus treated with this vapor phase produced some viscous oil droplets that aggregated on the cell monolayers. For this reason, we tried to expose PR8 to the vapor phase of the main compound present (49.1%) in the Chinese star anise, anethole. Treatment with the anethole vapor phase of the viral suspension, at the same condition described above, reduced the virus induced CPE (Figure 1), as confirmed by TCID_50_ assay: ≈82% reduction, logTCID_50_ = 2.25 vs. log TCID_50_ = 1.5, PR8-untreated vs. -treated, respectively. We can speculate that the most abundant components found in each vapor EO (limonene and β-pinene in *C. bergamia*, γ-terpinene in *M. alternifolia*, and 1,8-cineole in *E. globulus*) likely contribute to the vapor EOs efficacy against IAV. This hypothesis could also be confirmed by previously reported results describing the anti-influenza activity of 1,8-cineole [39] and limonene [40].

**Figure 2:**
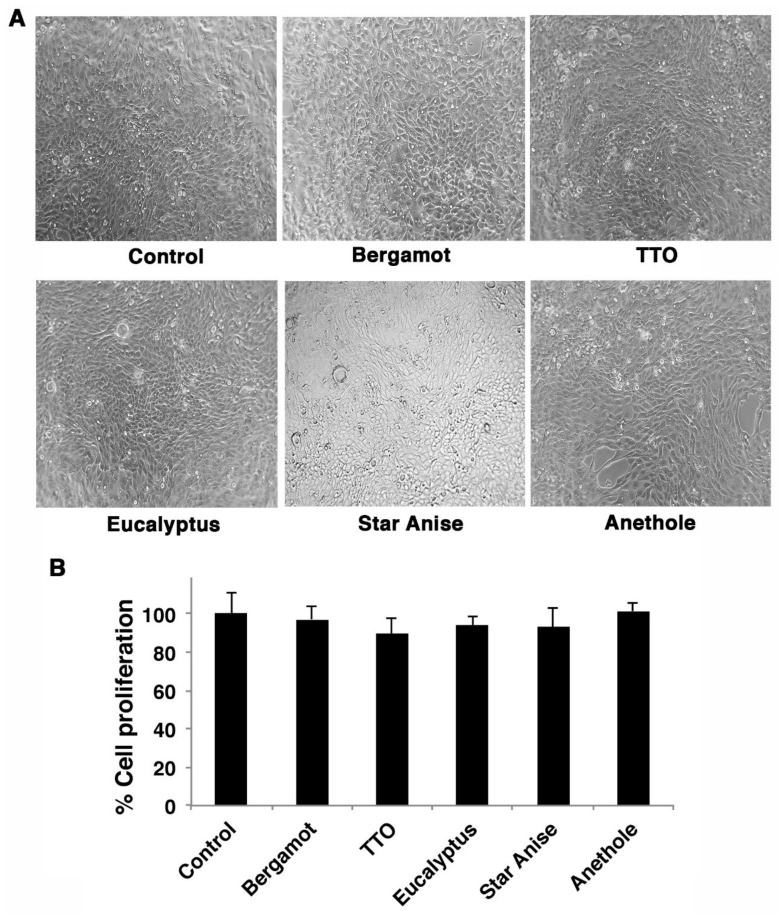
Cytotoxicity of EOs vapor phases. MDCK cell monolayers were treated with a mixture of PBS + EOs vapors and compared to cells incubated with PBS untreated (control). (**A**) Cell monolayers were observed by an inverted optical microscope or (**B**) evaluated by MTT assay. Cell viability was expressed as percentage (%) by comparison to control cells (considered as 100%). The values represent the mean ± S.D. of three technical replicates performed (*n* = 3).

To exclude that the observed antiviral effect of the EOs vapor phase was due to their cytotoxicity, cell monolayers were mock-infected with PBS treated with vapor EOs at the same conditions described above. After 24 h, the cells observed by an optical microscope showed no differences in morphology; the monolayers were confluent, and cells were not detached, indicating that the vapor EOs did not exert any cytotoxic effect (Figure 2A). Regarding the treatment with Chinese star anise, although the cell monolayer was still intact, this vapor phase produced some vacuoles probably due to droplets released on the vapor phase.

To confirm that effective vapor EOs were not toxic for the cells, MTT assay was per-formed on MDCK cell monolayers incubated with PBS treated with different vapor phases as described above. As shown in Figure 2B, the percentage of cell proliferation was comparable to cells not treated with vapor EOs and considered as control (100%).

**Figure 3:**
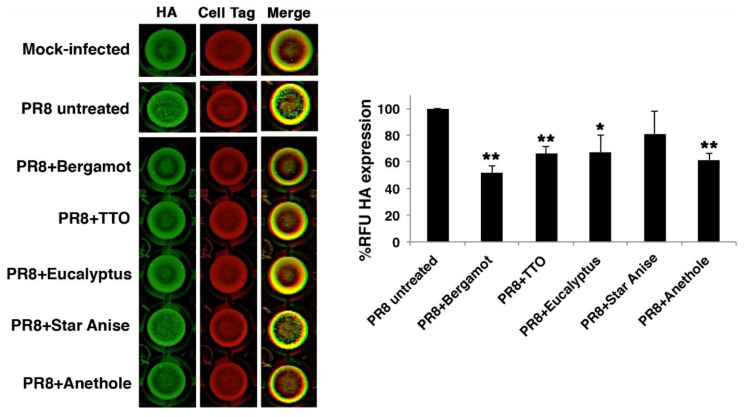
The expression of HA protein was analyzed on MDCK monolayers (**left panel**) by ICW assay using LI-COR Image Studio Software to measure the Relative Fluorescence Units (RFU) (**right panel**). The graph represents the percentage (%) of RFU calculated in cells infected with PR8 treated with different vapor EOs compared to that of PR8 untreated-infected cells (considered 100%). The values represent the mean ± S.D. of three technical replicates performed (*n* = 3). Statistical significance of data vs. PR8 untreated-infected cells was defined as * *p* < 0.05 and ** *p* < 0.001.

To confirm that the virucidal activity observed for vapor EOs (especially bergamot and TTO) led to reduced infectious ability, we chose to analyze viral hemagglutinin (HA) protein expression on MDCK cell monolayers infected with the mixture of PR8 treated or not with different vapor phases, by means of In Cell Western (ICW) assay (Figure 3, left panel). The analysis of HA protein expression (green) on infected cell monolayers indicated that HA was strongly reduced in cells infected with PR8 treated with bergamot or TTO compared to cells infected with PR8 untreated. As control of the integrity of cell monolayers, cells were confirmed by Cell Tag staining (red). In particular, the analysis of Relative Fluorescence Unit (RFU, right panel) showed a significant inhibition of HA protein expression (≈50% reduction) in cells infected with PR8 treated with bergamot vapor phase compared to that of PR8 untreated-infected cells (considered 100%) (Figure 3, right panel). As already observed in CPE and TCID_50_ assays, also, the mixture of PR8 plus TTO or eucalyptus was able to reduce viral infectivity.

### 2.3. Transmission Electron Microscopy (TEM) Imaging

It is well known that EOs, due to their lipophilic nature, can intercalate in the lipid bilayer of the viral envelope [41]; thus, membrane fluidity is altered, the viral envelopes are damaged and, in some cases, even broken [42]. As influenza viruses are enveloped by lipid membranes in which HA and neuraminidase (NA) viral surface glycoproteins are inserted, it is possible that EO vapors may act by non-specific intercalation in the lipid bilayer of the viral envelope, thus disorganizing the protein projections. To verify this hypothesis, the morphological integrity of virus particles was checked by negative staining transmission electron microscopy (TEM).

**Figure 4:**
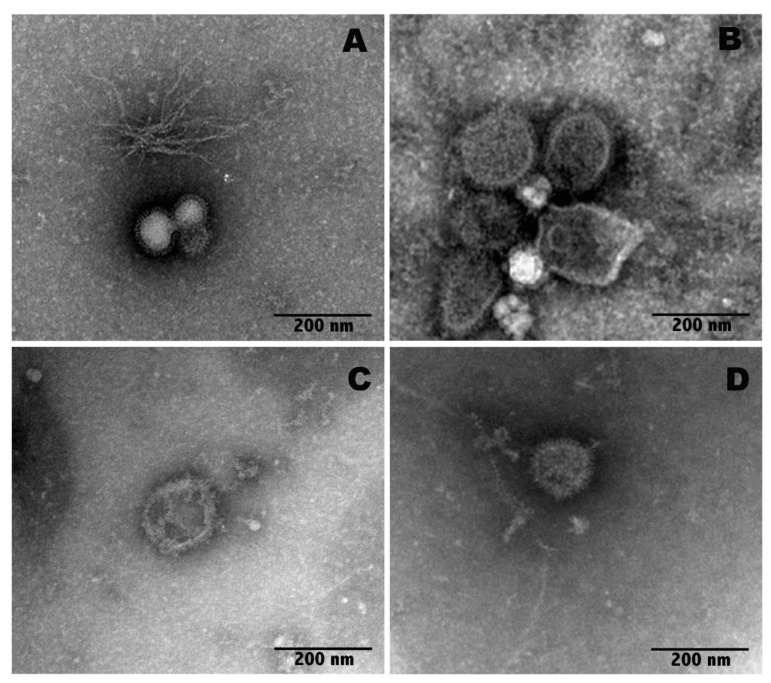
Negative staining of IAV particles. Control virions (**A**), bergamot (**B**), TTO (**C**) or anethole (**D**) vapor phases exposed virions.

The TEM images of the untreated IAV particles and the IAV particles after exposure to vapors of bergamot oil, TTO or anethole for 30 min are shown in Figure 4. Ultrastructural analysis showed that oil vapors directly affect the virions. In particular, after exposure to bergamot oil vapor, the IAV particles show a partially decomposed viral envelope, and exposure to the TTO vapor phase completely destroys the envelope, thus disorganizing the viral tips. In particular, using TEM imaging, it was possible to visualize the preservation of the morphology in the control viral particles (Figure 4A) and, at the same time, the structural changes in the viral particles following the treatments (Figure 4B–D). In particular, the particles exposed to vapors of bergamot, TTO or anethole appeared to expand in size.

As shown in Figure 4, the control virions display well-ordered surface tips (A); conversely, in the samples exposed to bergamot oil, the viral particles show a partially decomposed membrane and a disordered surface (B). In contrast, samples exposed to TTO were completely broken, and the tips appeared discontinuous (C). Although the virus particles exposed to anethole (D) were larger than the untreated virus particles, they appeared more preserved in morphology compared to the other treated samples (B, C).

In general, antiviral activity is classified into viral particle inactivation, adsorption inhibition and growth inhibition. The antiviral activity of EOs, which are lipophilic by nature, is probably due to the penetration into viral membranes, easily leading to membrane disintegration [39]. Basing on the results of the present study, it is very likely that there is a direct relationship between the lipophilic nature of the constituents of EOs and the viral envelope disruption. This mechanism of action has been demonstrated in a previous study on the herpes simplex virus type 1 (HSV-1), in which an ultrastructural alteration of the viral envelope by EOs has been observed by electron microscopy [43]. It has been observed that limonene and β-pinene (the main constituents of vapor phase of *C. bergamia*) and anethole (the most abundant component even in the vapor phase of *I. verum* EO) exhibited high anti-HSV-1 activity by direct interaction with free virus particles [40,44,45]. Thus, we may hypothesize that these compounds might directly interfere with influenza virion envelope structures or mask viral structures important for early steps of viral infection. A similar behavior, but less potent, was observed for 1,8-cineole (the most abundant component in the vapor phase of *E. globulus* EO) when added to HSV-infected cells prior to infection, even if plaque formation was reduced also after penetration of the viruses into the host cells [19]. Another study demonstrated that eucalyptus essential oil had no effect on adenovirus, a naked virus, confirming that EOs can affect viral infection by interfering with virion envelope structures [46]. Moreover, it was observed that γ-terpinene, the main constituent of vapor phase of *M. alternifolia*, shows only moderate antiviral effects when added to host cells prior to infection or after the entry of HSV into cells, exhibiting high anti-HSV-1 activity by the direct inactivation of free virus particles [19].

## 3. Materials and Methods

### 3.1. Materials

*Illicium verum* fruits (production lot number 02220202) and bergamot fruits were purchased from Salus in erbis (www.salusinerbis.it) and “Azienda Agricola Patea” (Brancaleone Marina, Italy), respectively. TTO (production lot number 192938014) and eucalyptus EO (production lot number 192938005) were obtained commercially from Caesar & Loretz GmbH (Hilden, Germany). Methanol was purchased from Sigma-Aldrich (Milano, Italy).

### 3.2. Gas Chromatography–Mass Spectrometry (GC–MS) Analysis

The analyses of the EOs were performed by a gas chromatograph with a flame ionization detector (FID) and coupled to a mass spectrometer (MS) Perkin Elmer Clarus 500 model (Waltham, MA, USA). The oven GC was equipped with a Varian Factor Four VF-1 capillary column and helium was used as carrier gas at a flow rate of 1 mL/min. The operative conditions followed [47] with some modifications.

Mass spectra were taken at 70 eV (EI) with a mass scan range of 40–500 *m*/*z*. The identification of constituents was obtained by matching their mass spectra with those stored in the Wiley and NIST 02 mass spectra libraries database. Furthermore, the Linear Retention Indices (LRIs), with reference to the series of C8–C30 aliphatic hydrocarbons, were calculated and compared with available retention data found in the literature. Relative amounts were expressed as percentages obtained by peak area normalization from GC-FID chromatograms without the use of an internal standard or correction factors. All analyses were performed three times.

### 3.3. Head Space GC/MS Analysis

To obtain the chemical characterization of the EOs vapor phase, a Perkin-Elmer Headspace Turbomatrix 40 (Waltham, MA, USA) autosampler connected to GC-MS was used. The analytical conditions such as temperature and equilibration time were optimized as previously reported [48]. For the sampling phase, about 1 mL of each EO was placed in 20 mL vials sealed with headspace PTFE-coated silicone rubber septa and caps. The quantification of identified compounds was performed by GC-FID as described in the previous section.

### 3.4. Cell Cultures

Madin–Darby canine kidney cells (MDCK) were acquired originally from ATCC (American Type Culture Collection, Rockville, MD, USA) and were passaged in cell culture flasks at 37 °C in a 5% CO_2_ atmosphere in Minimum Essential Medium Eagle (MEM, Sigma Aldrich, Milano, Italy) supplemented with 10% heat inactivated fetal bovine serum (FBS, Corning, NY, USA), glutamine 0.3 mg/mL, penicillin 100 U/mL and streptomycin 100 mg/mL.

### 3.5. Cell Toxicity Assays

The cytotoxicity of vapor EOs was studied after the observation of cell monolayers under an inverted optical microscope and by MTT proliferation assay [49]. In the MTT assay, MDCK cells were seeded in 96-well plates at a density of 2 × 10^4^ cells/well in 100 μL of complete MEM without phenol red for 24 h at 37 °C. Subsequently, cell monolayers were treated with PBS incubated with vapor phases and maintained at 37 °C. After 24 h, 10 μL of MTT solution (5 mg/mL) were added to each well for 3–4 h at 37 °C. Each sample was then acidified by adding 0.1 N HCl in isopropanol (100 μL/well) for 30 min under mild agitation to ensure the dissolution of all formazan crystals. The absorbance of samples was read at 570 nm, using an automatic plate reader (Multiskan EX, Ascent Software, Thermo Fisher Scientific, Waltham, MA, USA). The percentage (%) of cell proliferation in cells treated with EOs vapor was compared to that of control cells treated with PBS at the same conditions (considered 100%).

### 3.6. TCID_50_ (Tissue Culture Infectious Dose 50%) and Cytopathic Effect Assays

The virucidal activity of the vapor phase of the different EOs was tested by TCID_50_. The exposure of pure virus to vapor phases was performed with a modified method already described [31]. Briefly, 20 µL of human influenza virus A/Puerto Rico/8/34 H1N1 (PR8) was dried for 2 h on the underside of the caps from sterile Eppendorf 1.5 mL tubes. EOs (250 µL) were added to each tube and the caps, containing dried virus film, were replaced on the tube. The exposure of dried virus to different EOs vapor phases lasted for 30 min at 37 °C. Then, caps containing dried exposed film were removed from the tube, added to new tubes and reconstituted in 1 mL PBS. Each mixture was serially diluted and added (20 µL) to MDCK cell monolayers plated at 2 × 10^4^ cells/well in 96-well plates. Cells infected with serial dilutions (until 10^−4^) of reconstituted dried virus (8 replicates/dilution) were maintained at 37 °C in a 5% CO_2_ atmosphere for 1 h. After viral challenge, the inoculum was removed, and cells were incubated in culture medium supplemented with 2% heat-inactivated FBS.

The Cytopathic Effect (CPE) induced by the infection was evaluated 24 h post infection and TCID_50_ was calculated using the Reed–Muench method, as previously reported [50]. Dried virus, not exposed to any oil, was diluted in PBS and used as control. No viral infectivity loss due to drying was detectable by TCID_50_.

### 3.7. In-Cell Western (ICW) Assay

The viral protein expression was analyzed by ICW assay, as already described [11]. Briefly, MDCK confluent cell monolayers were infected with PR8 treated or not with EOs vapor phases for 1 h at 37 °C. After viral adsorption, cells were washed with PBS, supplemented with fresh medium plus 2% FBS and maintained for 24 h at 37 °C. Cell monolayers were then fixed with 3.7% formaldehyde for 20 min, permeabilized with 0.1% Triton X-100 and incubated with Odyssey blocking buffer (LI-COR Bioscience, Lincoln, NE, USA) for 60 min at room temperature. The cells were stained at 4 °C overnight with primary antibodies against influenza HA protein. After incubation, three washes with PBS plus 0.1% Tween 20 were performed, and then, the cells were stained with a mixture of fluorochrome-conjugated secondary antibodies (fluorescence emission at 800 nm), properly diluted in Odyssey blocking buffer and fluorochrome-conjugated Cell Tag (fluorescence emission at 700 nm), for 1 h at room temperature. Cell Tag was used as control of the integrity of cell monolayer. Subsequently, three washes with PBS plus 0.1% Tween 20 were performed, and plates were analyzed by the Odyssey infrared imaging system (LI-COR). Integrated intensities of fluorescence were determined by the LI-COR Image Studio software, and the relative fluorescence unit (RFU) was expressed as percentage compared to untreated infected cells (100%).

### 3.8. Transmission Electron Microscopy (TEM) Imaging

For TEM visualization, after 30 min exposure to EOs vapors, unexposed (control) or exposed viral particles were resuspended in PBS. A drop of each sample (10 μL) was absorbed onto carbon-formvar-coated, 400-mesh EM copper grids. Negative staining was carried out by utilizing 2% phosphotungstic acid (PTA) (pH 7.0) for 30 s, and samples were examined using an EM 208 FEI transmission electron microscope operated at 80 kV. Post-imaging manipulation was performed in Photoshop (Adobe, San Jose, CA, USA) and was limited to image cropping and equal adjustments of image levels.

## 4. Conclusions

In conclusion, we analyzed the EOs and their vapor phase chemical compositions by the head-space gas chromatography-mass spectrometry technique. *Citrus bergamia* and *M. alternifolia* essential oil vapors showed interesting anti-influenza A virus activity following 30 min exposure. Under these conditions, the vapors showed no measurable adverse effect on epithelial cell monolayers. It was suggested from the results of the present study that the essential oil inactivates directly the viral particles. Indeed, it was reported that limonene, β-pinene, and anethole directly interact with free HSV-1 particles. Thus, we may hypothesize that these compounds might directly interfere with influenza virion envelope structures or mask viral structures important for early steps of viral replication. A similar behavior, but less potent, was observed for 1,8-cineole (the most abundant component in the vapor phase of *E. globulus* EO) when added to HSV-infected cells prior to infection, even if plaque formation was reduced also after penetration of the viruses into the host cells. Moreover, γ-terpinene, the main constituent of the vapor phase of *M. alternifolia*, shows only moderate antiviral effects when added to host cells prior to infection or after entry of HSV into cells, exhibiting high anti-HSV-1 activity by the direct inactivation of free virus particles. Furthermore, as both IAV and HSV-1 are surrounded by an envelope, these results could be useful for further studies on other enveloped viruses. In addition, *E. globulus* vapor EO reduced viral infection by 78% with no cytotoxicity, while *I. verum* was not effective. Therefore, we tested the most abundant component in the vapor phase of *I. verum* EO, anethole, finding that it reduced viral infection by 82%. To deepen the mechanism of action, the morphological integrity of virus particles was checked by negative staining transmission electron microscopy, showing that, probably due to their lipophilic nature, they penetrate the lipid bilayer of the viral envelope, damaging the membranes or leading to their complete destruction. These ultrastructural results support the use of EOs as effective virucidal agents enveloped viruses.

Taken together, our data suggest that these EOs vapor phases possess effective anti-influenza virus activity under conditions that did not adversely affect cultured epithelial cells. The viral envelope appeared to be a major target. Thus, some of these oil vapors could be potentially useful to reduce the viral infectivity and virus contamination of the environment.

## Figures and Tables

**Table 1:** Chemical composition (percentage mean value ± standard deviation) of EOs.

N°	Component ^1^	LRI ^2^	LRI ^3^	BEO ^4^ *(*%)	IV-EO ^5^ *(*%)	TTO ^6^ (%)	EEO ^7^ (%)
1	α-thujene	920	923	0.3 ± 0.03	-	-	1.7 ± 0.02
2	α-pinene	941	943	1.3 ± 0.02	0.3 ± 0.03	0.2 ± 0.02	-
3	sabinene	973	972	-	-	tr	-
4	β-myrcene	981	983	1.5 ± 0.03	tr	0.1 ± 0.00	0.6 ± 0.02
5	β-pinene	988	986	7.3 ± 0.02	-	0.1 ± 0.00	-
6	α-phellandrene	1008	1005	-	0.2 ± 0.02	tr	0.5 ± 0.02
7	α-terpinene	1013	1010	-	-	1.1 ± 0.02	-
8	p-cymene	1020	1016	0.7 ± 0.02	tr	0.3 ± 0.04	1.7 ± 0.01
9	limonene	1021	1023	31.9 ± 0.06	0.1 ± 0.02	-	-
10	1,8-cineole	1026	1025	-	-	0.5 ± 0.02	93.7 ± 0.02
11	cis-β-ocimene	1035	1032	0.5 ± 0.02	-	-	-
12	β-terpinene	1040	1036	-	tr	-	-
13	trans-β-ocimene	1044	1043	0.2 ± 0.02	-	-	-
14	trans-sabinene hydrate	1057	1053	-	0.2 ± 0.02	-	-
15	γ-terpinene	1059	1054	7.5 ± 0.02	-	2.7 ± 0.03	1.6 ± 0.02
16	cis-sabinene hydrate	1072	1069	-	tr	-	-
17	terpinolene	1079	1080	-	-	tr	tr
18	linalool	1090	1092	17.0 ± 0.03	1.0 ± 0.02	-	-
19	terpinen-4-ol	1164	1160	-	-	92.4 ± 0.03	tr
20	estragole	1180	1177	-	3.2 ± 0.02	-	-
21	α-terpineol	1185	1183	tr	-	0.6 ± 0.02	0.1 ± 0.00
22	carveol	1210	1201	tr	-	-	-
23	cis-piperitol	1219	1215	-	-	tr	--
24	p-anisaldehyde	1232	1229	-	0.2 ± 0.03	-	-
25	linalyl acetate	1259	1252	31.4 ± 0.02	-	-	-
26	anethole	1269	1260	-	93.0 ± 0.07	-	-
27	α-citral	1291	1287	0.1 ± 0.02	-	-	-
28	α-copaene	1373	1370	-	-	0.1 ± 0.00	-
29	β-elemene	1410	1406	-	-	tr	-
30	β-caryophyllene	1426	1424	-	0.7 ± 0.03	0.1 ± 0.00	-
31	α-bergamotene	1435	1430	-	tr	-	-
32	aromadendrene	1459	1460	-	tr	0.4 ± 0.03	-
33	humulene	1470	1465	-	tr	tr	-
34	γ-muurolene	1490	1486	-	-	0.2 ± 0.02	-
35	ledene	1499	1492	-	-	0.5 ± 0.02	-
36	β-bisabolene	1501	1501	0.1 ± 0.02	tr	-	-
37	α-farnesene	1510	1506	0.1 ± 0.01	tr	-	-
38	δ-cadinene	1528	1530	-	-	0.4 ± 0.02	-
39	spathulenol	1573	1571	-	-	tr	-
40	viridiflorol	1580	1583	-	-	0.1 ± 0.01	-
41	globulol	1596	1594	-	-	0.1 ± 0.01	-
42	cubenol	1635	1631	-	-	0.1 ± 0.01	-
	SUM			99.9	98.9	100.0	99.9
	Terpenoids			68.3	1.8	98.0	99.9
	Sesquiterpenoids			0.2	0.7	1.9	-
	Other			31.4	96.4	0.1	-

^1^ The components are reported according to their elution order on apolar column; ^2^ Linear Retention Indices measured on apolar column; ^3^ Linear Retention indices from literature; ^4^ Percentage mean values of *C. bergamia* liquid phase components; ^5^ Percentage mean values of *I. verum* liquid phase components; ^6^ Percentage mean values of *M. alternifolia* liquid phase components; ^7^ Percentage mean values of *E. globulus* liquid phase components; - Not detected; tr: traces (mean value <0.1%).

**Table 2:** Chemical composition of volatiles (percentage mean value ± standard deviation) of EOs.

N°	Component ^1^	LRI ^2^	LRI ^3^	BEO ^4^ (%)	IV-EO ^5^ (%)	TTO ^6^ (%)	EEO ^7^ (%)
1	α-thujene	920	923	1.5 ± 0.02	0.4 ± 0.02	3.5 ± 0.04	5.6 ± 0.02
2	α-pinene	941	943	6.5 ± 0.02	19.1 ± 0.06	10.0 ± 0.04	-
3	camphene	950	946	0.3 ± 0.02	0.3 ± 0.03	0.1 ± 0.00	-
4	sabinene	974	972	-	-	0.3 ± 0.02	-
5	β-myrcene	981	983	3.9 ± 0.03	2.3 ± 0.04	1.5 ± 0.04	0.8 ± 0.00
6	β-pinene	988	986	20.4 ± 0.05	-	1.7 ± 0.06	-
7	α-phellandrene	1008	1005	0.2 ± 0.01	5.9 ± 0.07	1.4 ± 0.01	0.6 ± 0.01
8	α-terpinene	1013	1010	-	-	18.1 ± 0.02	-
9	p-cymene	1020	1016	1.1 ± 0.03	0.5 ± 0.03	10.7 ± 0.04	1.9 ± 0.04
10	limonene	1021	1023	51.2 ± 0.03	3.8 ± 0.06	-	-
11	1,8-cineole	1026	1025	-	-	8.3 ± 0.03	89.8 ± 0.03
12	cis-β-ocimene	1035	1032	0.7 ± 0.03	0.4 ± 0.05	-	-
13	β-terpinene	1040	1036	-	0.7 ± 0.03	-	-
14	trans-β-ocimene	1044	1043	0.3 ± 0.04	-	-	-
15	γ-terpinene	1059	1054	9.2 ± 0.04	2.7 ± 0.03	29.3 ± 0.03	1.1 ± 0.02
16	cis-sabinene-hydrate	1072	1069	-	5.0 ± 0.05	-	-
17	terpinolene	1079	1080	-	-	4.6 ± 0.04	tr
18	linalool	1090	1092	4.6 ± 0.02	3.9 ± 0.05	-	-
19	terpinen-4-ol	1164	1160	0.1 ± 0.02	-	10.2 ± 0.05	-
20	estragole	1180	1177	-	5.5 ± 0.06	-	-
21	α-terpineol	1185	1183	-	-	0.3 ± 0.04	0.2 ± 0.01
22	anethole	1269	1260	-	49.1 ± 0.05	-	-
23	α-farnesene	1510	1506	-	0.3 ± 0.02	-	-
	SUM			100.0	99.9	100.0	100.0
	Terpenoids			100.0	45.0	100.0	100.0
	Sesquiterpenoids			-	0.3	-	-
	Other			-	54.6	-	-

^1^ The components are reported according to their elution order on apolar column; ^2^ Linear Retention Indices measured on apolar column; ^3^ Linear Retention indices from the literature; ^4^ Percentage mean values of *C. bergamia* vapor phase components; ^5^ Percentage mean values of *I. verum* vapor phase components; ^6^ Percentage mean values of *M. alternifolia* vapor phase components; ^7^ Percentage mean values of *E. globulus* vapor phase components; - Not detected; tr: traces (mean value <0.1%).

## Data Availability

Data is contained within the article.
